# Placenta previa percreta left *in situ *- management by delayed hysterectomy: a case report

**DOI:** 10.1186/1752-1947-5-418

**Published:** 2011-08-25

**Authors:** Minna Tikkanen, Vedran Stefanovic, Jorma Paavonen

**Affiliations:** 1 Helsinki University Hospital, Department of Obstetrics and Gynecology, Helsinki, Finland

## Abstract

**Introduction:**

Placenta percreta is an obstetric emergency often associated with massive hemorrhage and emergency hysterectomy.

**Case presentation:**

We present the case of a 30-year-old African woman, gravida 7, para 5, with placenta percreta managed by an alternative approach: the placenta was left *in situ*, methotrexate was administered, and a delayed hysterectomy was successfully performed.

**Conclusions:**

Further studies are needed to develop the most appropriate management option for the most severe cases of abnormal placentation. Delayed hysterectomy may be a reasonable strategy in the most severe cases.

## Introduction

Placenta accreta (PA) is characterized by abnormal invasion of the placenta into the myometrium. PA is defined as superficial invasion, placenta increta as middle layer invasion and placenta percreta as deep invasion, which is the most severe form of PA with an incidence of one in 7000. All three types are collectively known as placenta accreta. The incidence of PA has dramatically increased due to increasing Caesarean section rates [1,2]. Although rare, PA is one of the most severe pregnancy complications. Maternal morbidity and mortality associated with PA is mainly caused by massive obstetric hemorrhage or emergency hysterectomy, and PA is often diagnosed during delivery or immediately post-partum leading to an obstetric emergency [1,3,4]. Studies suggest that antenatal diagnosis may reduce obstetric hemorrhage-related morbidity [5,6]. Furthermore, in some cases a morbidly adherent PA can be left *in situ *[7,8]. Such conservative management may allow delayed removal of the placenta to avoid massive hemorrhage during an attempted forced removal of the adherent placenta. We describe a case in which placenta percreta was left *in situ*. Subsequent post-partum hemorrhage was successfully managed by delayed hysterectomy.

## Case presentation

**Figure 1:**
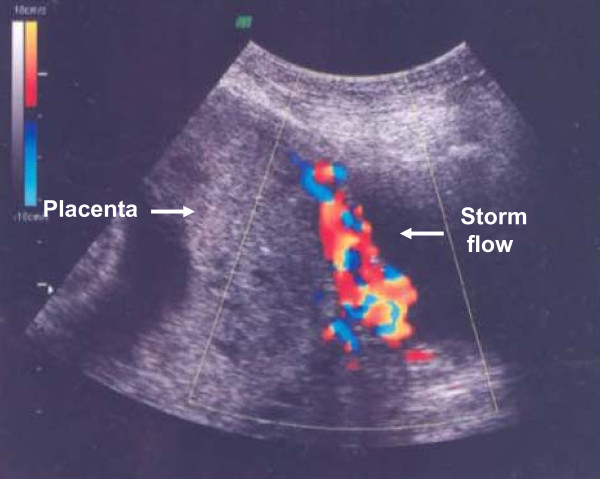
**Ultrasonography findings at gestational week 29 consistent with placenta accreta**.

**Figure 2:**
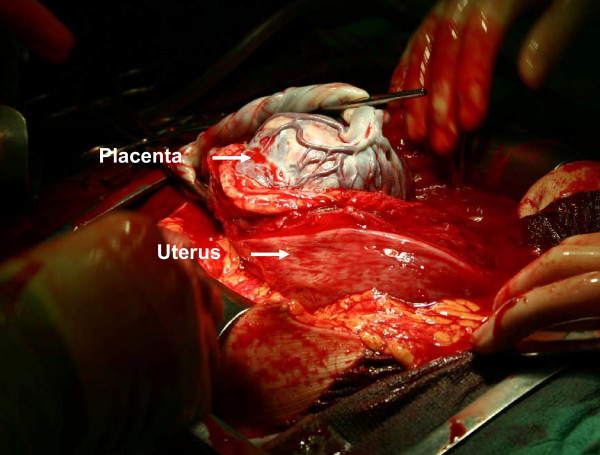
**Operative findings during Caesarean section**. Note placenta previa associated with placenta accreta suggestive of placenta percreta.

**Figure 3:**
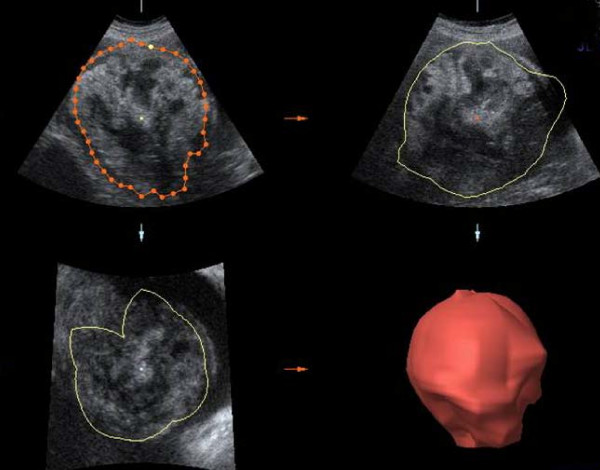
**Ultrasonographic findings of placenta accreta left *in situ *with measurements to estimate the total volume during follow-up**.

**Figure 4:**
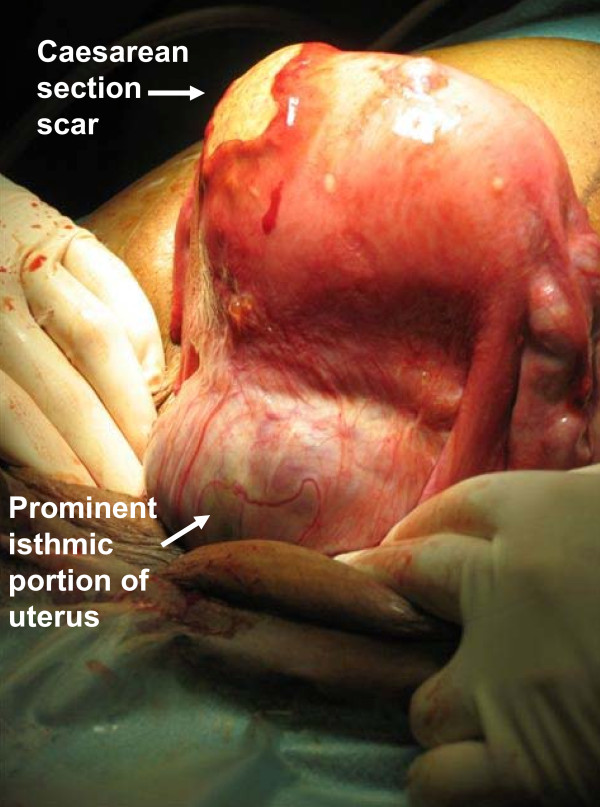
**Uterine findings during laparotomy**. Note Caesarean section scar at uterine fundus and bulging isthmic part with placenta accreta/percreta.

**Figure 5:**
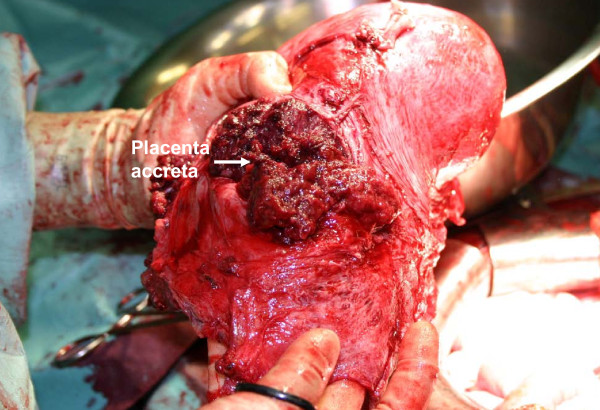
**Hysterectomy specimen opened**. Note placenta previa percreta left *in situ*.

**Figure 6:**
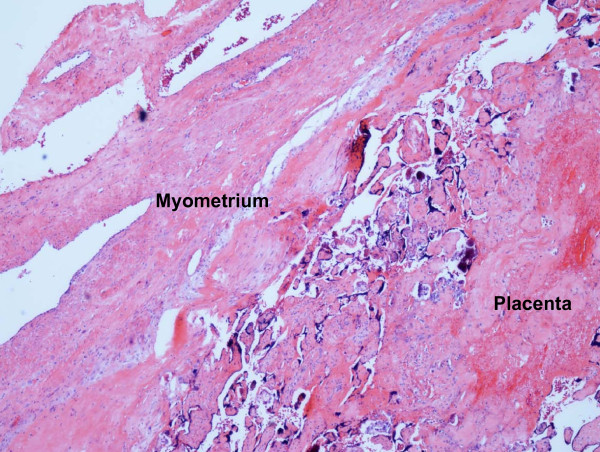
**Histopathological specimen consistent with placenta percreta (magnification 40 ×)**.

Our patient was a 30-year-old African woman, gravida 7, para 5. Her second screening ultrasound at 21 weeks of gestation showed normal fetal anatomy and placenta previa. She was referred in her 28th gestational week from her antenatal clinic to the University Hospital Outpatients Maternity Clinic because of anemia and mild thrombocytopenia. The first suspicion of placenta accreta or percreta based on pathological 'storm flow' on Doppler ultrasound was raised at 29 gestational weeks (Figure 1). Severe placenta accreta was confirmed at 35 gestational weeks both by ultrasonography and MRI. As a premature elective delivery was anticipated, she underwent amniocentesis during the same examination. Her amniotic fluid erythropoietin level was normal and her amniotic fluid lamellar body count was 3, consistent with an immature fetal lung maturity profile. Intra-muscular betamethasone was administered using two standard doses of 12 mg 24 hours apart. An elective Caesarean section was performed 10 days later (Figure 2) with simultaneous tubal ligation. The weight of the healthy newborn boy was 3.1 kg. The Caesarean section was performed under general anesthesia due to mild thrombocytopenia. A classical uterine incision was used for extraction of the fetus in order to avoid the morbidly adherent low placenta. The internal iliac arteries were catheterized pre-operatively via insertion of balloons to decease peri-operative bleeding. The placenta was left *in situ *because of placenta previa with severe placenta percreta. Bilateral uterine artery embolization was performed. The total amount of blood loss during the Caesarean section was 300 mL. Our patient then received a single 100 mg dose of intra-muscular methotrexate. She recovered uneventfully with no complications, and was discharged on the seventh day post-partum. She received prophylactic metronidazole and cephalosporin orally for five days. Our patient returned to the emergency room the next day because of low abdominal pain. Her C-reactive protein (CRP) concentration was 45 g/L. A pelvic examination showed no significant findings. She again returned to the emergency room a week later because of recurrent low abdominal pain. Her CRP level was 20 g/L and chorionic gonadotropin (hCG) concentration was 1828U. Serial periodic serum hCG levels decreased from 1492U to 848U to 562U to 200U over the first four weeks, respectively. Her total intra-uterine placental volume was measured by repeat ultrasound examinations. The placenta shrunk from 900 cm^3 ^on the first examination to 630 cm^3 ^and then to 319 cm^3 ^over four weeks (Figure 3). Ultrasound examination at four weeks showed necrotic areas within the placenta left *in situ *with heavily vascularized areas next to the bladder and a strong storm flow phenomenon. Our patient again returned to the emergency room three weeks later (seven weeks from the Caesarean section) because of heavy uterine bleeding. Her general condition was satisfactory, and she received three units of red blood cells and was hospitalized at this time. Her bleeding continued, became more profuse and her condition became unstable, so a decision to perform an emergency laparotomy was made. A laparotomy with emergency hysterectomy was then performed. Her uterus was still relatively large with prominent and bulging isthmic portion (Figure 4). The total blood loss during the hysterectomy was 3.7 L. Her uterus was filled by hematoma and placenta previa with deep myometrial invasion (Figure 5). A histopathological examination showed placenta percreta with chorioamnionitis, dilated blood vessels and incomplete involution of the uterus (Figure 6). Our patient recovered uneventfully. She was discharged on the fifth post-operative day in good condition.

## Discussion

PA causes considerable maternal morbidity and mortality and is the major indication for emergency peri-partum hysterectomy. Antenatal confirmation of PA diagnosis is often difficult [3,9,10]. The management is usually an elective cesarean delivery and hysterectomy, but this approach often causes massive hemorrhage and may cause injury of adjacent organs due to the morbidly adherent placenta [8]. Delayed trans-vaginal removal of the placenta has also been described [11]. Some studies suggests that leaving placenta *in situ *lowers the risk for subsequent hysterectomy and may hence be an option in cases when emergency hysterectomy is considered too risky or fertility needs to be preserved [7,8,12].

We describe a severe case of PA histopathologically defined as placenta percreta in which the placenta was left *in situ*. Subsequently, our patient had multiple emergency room visits and ultimately developed severe post-partum hemorrhage leading to delayed emergency hysterectomy. This case suggests that in some cases with placenta accreta and percreta, leaving placenta *in situ *may be an alternative management option allowing delayed hysterectomy. This management option may be safer than primary hysterectomy since delayed hysterectomy may be easier to perform than emergency hysterectomy immediately post-partum (due to placental involution and decreased uterine vascularity). We are currently developing a management algorithm for women with an antenatal diagnosis of placenta percreta in which the placenta is left *in situ*, combined with parenteral methotrexate and elective delayed hysterectomy (although methotrexate is not used in all maternal-fetal centers in such clinical situations). This is justified based on our experience of other cases of placenta accreta in which the placenta was left *in situ*. Total placental involution with no complications occurred in only one out of five such cases with the placenta left *in situ*.

## Conclusions

Placenta percreta is an obstetric emergency often associated with massive hemorrhage leading to emergency hysterectomy. We describe a severe case of placenta percreta in which the placenta was left *in situ*, methotrexate administered and a delayed hysterectomy successfully performed. Delayed hysterectomy may be a reasonable management strategy in the most severe cases.

## Consent

Written informed consent was obtained from the patient for publication of this case report and any accompanying images. A copy of the written consent is available for review by the Editor-in-Chief of this journal.

## Competing interests

The authors declare that they have no competing interests.

## Authors' contributions

JP and MT analyzed and interpreted the data from our patient, VS obtained the figures, JP drafted the manuscript, and JP, MT and VS revised the manuscript for important intellectual content. All authors read and approved the final manuscript.
